# Nurses' experiences of person‐centred care planning using video‐conferencing

**DOI:** 10.1002/nop2.1452

**Published:** 2022-11-03

**Authors:** Ann‐Therese Hedqvist, Ann Svensson, Lena G. Larsson

**Affiliations:** ^1^ Linnaeus University Kalmar Sweden; ^2^ Region Kalmar Västervik Sweden; ^3^ School of Business, Economics and IT University West Trollhättan Sweden; ^4^ Department of Health Sciences University West Trollhättan Sweden; ^5^ Research, Education, Development and Innovation, Primary Health Care Region Västra Götaland Gothenburg Sweden

**Keywords:** care planning, collaboration, frail older persons, nurses, person‐centred care, video conferencing

## Abstract

**Aim:**

The aim was to illuminate how nurses experience person‐centred care planning using video conferencing upon hospital discharge of frail older persons.

**Design:**

Care planning via video conferencing requires collaboration, communication and information transfer between involved parties, both with regard to preparing and conducting meetings. Participation of involved parties is required to achieve a collaborative effort, but the responsibilities and roles of the involved professions are unclear, despite the existence of regulations.

**Method:**

A qualitative content analysis was conducted based on 11 individual semi‐structured interviews with nurses from hospitals, municipalities and primary care in Sweden.

**Results:**

This study provides valuable insights into challenges associated with care planning via video conferencing. The meeting format, that is video conferencing, is perceived as a barrier that makes the interaction challenging. Shortcomings in video technology make a person‐centred approach difficult. The person‐centred approach is also difficult for nurses to maintain when the older person or relatives are not involved in the planning.

## 1 INTRODUCTION

Frail older persons are recognized as an especially vulnerable patient group. They are dependent on coordinated and integrated healthcare efforts, provided by healthcare professionals from hospitals, primary care and municipal care. The increasing number of older persons with complex illnesses, and shorter inpatient care, calls for provision of care closer to home and emphasizes the need for collaboration between healthcare providers (Dahl et al., 2014), especially in connection with discharge from inpatient care, which is regulated by law in Sweden (SFS 2017:612).

Discharge from hospital implies a transition of care from one site to another. Such a transition can make the transferred persons extra vulnerable, especially frail older persons with multiple health problems, and can result in fragmented care, medication errors, medical complications and dissatisfaction among professionals, patients or their relatives (Elliott et al., 2018; Newnham et al., 2017). Therefore, a coordinated individual care plan (CIP) is utilized to plan and unite coordinated efforts at home in order for the frail older person to leave the hospital in a safe manner. Thus, a coordinated care‐planning meeting, initiated by the nurses at the primary healthcare centre, takes place prior to discharge.

Coordinated care planning can provide opportunities to capture the frail older person's need for care, and support coordinated care planning efforts. The care‐planning meeting involves the patient, relatives and professionals from different healthcare providers, and aims to plan and transfer information regarding care. However, the nurses are the main professional group in the care‐planning meeting. The care planning should be done in such a way that communication is efficient and safe (Chichirez & Purcărea, 2018), and the older person receives care of high quality, continuity and security (Svensson et al., 2016). However, there appears to be a lack of trust between the different parties involved (Larsen et al., 2017; Larsson et al., 2019), similar to what has been observed in person‐centred care (Forsman & Svensson, 2019), since deficits in collaboration and communication are common (Nordmark et al., 2016). Thus, coordination between hospitals, primary care and municipal care is an important issue in the healthcare of frail older persons with complex care needs (Kneck et al., 2019).

As an approach to conduct care‐planning meetings, video conferencing is now used increasingly often. A growing number of functions in the healthcare system are being performed digitally (Westerlund & Marklund, 2020) as video technology is employed in telemedicine technology (Baker & Stanley, 2018; Ignatowicz et al., 2019), teleconsultations (Diedrich & Dockweiler, 2021) and virtual care delivery (Li et al., 2021) such as pain management programs (Walumbe et al., 2021). Video technology can improve the efficiency of care and increase the access to specialist expertise (Nilsson et al., 2010). The Covid‐19 pandemic has demonstrated the possibilities and importance of video technology in healthcare. Almost all types of meetings can now be conducted using video conferencing, partly as a result of visitor limitations during the pandemic. The scope of this study is limited to care‐planning meetings using video conferencing in which multiple healthcare professionals meet the patient and his/her family together. While the use of video conferencing in care‐planning meetings has not been investigated to a great extent, research shows that healthcare professionals are generally positive to using the technology in this context (Newbould et al., 2017; Shubber et al., 2018). Pols (2012) argues that technology can offer adequate alternatives to personal communication since physical contact should not be necessary in meetings, instead, the health and healthcare context should be in focus. Hence, video conferencing creates new opportunities, but also new aspects to consider, since there are challenges in digitalization of healthcare (Allwood, 2017). In order to avoid dehumanization in an increasingly digitized care setting, it is important to promote relationships and consider the older persons' autonomy and dignity (Jacobs et al., 2017). A person‐centred approach is not always easy to maintain, as healthcare meetings via video conferencing seem to imply some sort of barrier between people (Hedqvist & Svensson, 2019). Understanding of how technology and video conferencing can support and improve healthcare for older people is lacking (Beirao et al., 2016; Ignatowicz et al., 2019). Moreover, extant literature recounts an extensive need for increased involvement of patients in the dialogue about health conditions, and calls for preparation for the care after discharge (Forsman & Svensson, 2019; Lindblom et al., 2020). Furthermore, aspects related to the nurses' experiences and the person‐centred care presented to patients, need to be considered. It is also important to understand the potential benefits, limitations and professional implications that affect the adoption and use of video conferencing (Penny et al., 2018). Therefore, further studies are required to understand nurses' experiences of how older persons' care needs can be coordinated by using video conferencing, while at the same time maintaining a person‐centred approach at a physical distance. The aim of this paper is to illuminate how nurses experience person‐centred care planning using video conferencing upon hospital discharge of frail older persons.

## 2 THEORETICAL BACKGROUND

Hospital discharge, that is, the transition of frail older persons from hospital to their homes, requires coordination of care planning between different healthcare providers. Care planning using video conferencing is increasing, while care planning during in‐person meetings is decreasing. At the same time, video conferencing implies challenges for person‐centred care.

### 2.1 Coordination of care planning

To provide good healthcare to frail older persons with multiple illnesses, interprofessional collaboration is necessary (Ekdahl et al., 2010; Ivanoff et al., 2018). This implies an active and ongoing partnership between people from distinct professional cultures, as well as from different healthcare providers. Collaboration requires that professionals, and nurses in particular, work together to solve problems and provide common healthcare efforts (Donnelly et al., 2021; Pullon et al., 2016). Coordination between different healthcare providers such as hospitals, primary care and municipal care, is thus an important prerequisite for providing functioning healthcare to frail older persons with complex care needs. Timely information sharing among healthcare providers is a necessary part of the coordination process. However, the healthcare that older persons receive from multiple providers is often unorganized and confusing (Elliott et al., 2018). The coordination can be adversely affected by lack of trust between different healthcare providers (Larsson et al., 2019). Interaction and interpersonal relationships emerge as central aspects in well‐functioning coordination and care‐planning meetings that focus on older persons' healthcare and discharge from hospital (Larsson et al., 2017; Petersen et al., 2019). Thus, high demands are placed on nurses in the collaboration and coordination processes, as well as in care‐planning meetings, to promote shared decision‐making regarding the older persons' care among the healthcare professionals as well as the patient and relatives (Hansson et al., 2018). Thus, patient engagement processes are also important in order to enhance appropriate coordination for the best interests of the older person.

Personnel from relevant healthcare professions, with skills needed for meeting the older persons' need for healthcare and social care efforts after discharge, participate in the care‐planning meetings. Today, video conferencing is a widely used alternative to in‐person meetings for care planning, partly due to the Covid‐19 pandemic (Liu et al., 2021). Video conferencing is the technology that is considered most similar to in‐person meetings (Park et al., 2014).

### 2.2 Care planning via video conferencing

Video conferencing makes it possible for professionals, such as primary healthcare nurses, municipal nurses, municipal assistance officer, rehabilitation professionals from the primary healthcare centre and from the municipality, and others, to attend a meeting at the same time together with the patient (Larsson et al., 2019). This is considered positive for the frail older person as it provides an opportunity for people with different professions to meet, and it enables personalized care planning before discharge from the hospital (Hofflander, 2015). Video conferencing also enables relatives to participate in the care planning even if they live far away or cannot go to the hospital. Studies show that video‐conference meetings generally take less time than in‐person meetings and tend to be more well‐structured. In addition to the meetings being shorter and more efficient, travel times also decrease dramatically (Nordmark et al., 2016; Vollenbroek‐Hutten et al., 2017). A study on nurses' attitudes towards care planning via video conferencing shows that their overall attitude towards using this technology for meetings is positive, especially as it increases efficiency (Shubber et al., 2018). There are, however, some concerns in the literature among nurses that frail older persons may be overridden in video‐conference meetings. One of the most difficult aspects of having a video‐conference meeting is to make sure that all participants are aware of, and involved in, what is happening (Marlow et al., 2016). Nurses should include the older person and relatives in the video‐conference meeting (Graves & Doucet, 2016). However, older persons and relatives often feel excluded in the meetings (Hedqvist et al., 2020). Human contact, touch and non‐verbal behaviour are presented as very important parts of the care, parts which are jeopardized by the use of video conferencing (Miller, 2010).

Video conferencing enables meetings to take place without the requirement of being physically present in the same room (Ignatowicz et al., 2019). The performance of the video‐conference system, which is based on Internet connectivity, image and sound quality, strongly influences the meeting experience, as limited image quality as well as small screen images hinder communication (Allen et al., 2008). Delays in sound transmission adversely affect the conversation and communication as it becomes difficult to maintain a natural flow and take turns in the conversation. Studies show that it can be more difficult to establish and maintain trust in each other in a video‐conference meeting, compared to face‐to‐face. Thus, there are several challenges associated with video‐conference meetings, and video conferencing does not always live up to its expectations. In fact, the implementation and use of video conferencing in care planning have proven to be more complex and time consuming than initially anticipated (Shubber et al., 2018). In video‐conference meetings, challenges can increase further with the number of participants. Communication through video conferencing is perceived to be more difficult than it would be face‐to‐face, and impairs the ability to build trust with the patient (Graves et al., 2018). Factors that determine the quality of a video‐conference meeting are related to the possibilities of establishing human interaction, where eye contact is an important aspect.

### 2.3 Person‐centred care via video conferencing

Over the past two decades, person‐centred care has gained more attention, especially in relation to research and policies linked to high‐quality health care (Mead & Bower, 2000). Currently, there is no uniform definition of the concept of person‐centred care, but a recurring theme concerns the ethical issue of treating patients as persons (Epstein & Street, 2011). A prerequisite for performing person‐centred care is the healthcare provider's ability to communicate and interact with the patient in a person‐centred manner. In providing health care, professionals are required to be open and responsive to each patient, perceive the patient as an expert, with regard to his/her own health, and treat the patient as a partner and an equal person.

Previous literature has found that person‐centred care has a positive impact on healthcare outcomes (Olsson et al., 2013). Person‐centred care, which involves shared decision‐making, aided decision‐making and meaningful encounters, gives frail older persons the possibility to confirm and retain a position in the context, and increases their well‐being and independence (Forsman & Svensson, 2019). Video conferencing specifically challenges person‐centred care, as the professionals encounter a work phenomenon that entwines task execution and relationship building within a virtual media environment (LeRouge et al., 2012).

Person‐centred meetings require knowledge and ability to understand the patient's experiences and needs through a conscious presence created through interaction, communication and reciprocity (McCormack & McCance, 2006). Person‐centred meetings via video conferencing thus entail new challenges, which require healthcare professionals to learn how to create and maintain good interactions and caring relationships via virtual meetings. Person‐centred meetings via video conferencing focus specifically on the patient's ability to fully engage in the care, and the care that needs to be planned. The patient's mental status can affect the success of person‐centred meetings. Video conferencing involves a risk of losing social phenomena. Small expressions or movements may not be noticed or become blurred or diminished in the virtual meeting. Some people can also feel uncomfortable with the lack of physical contact. Moreover, older persons with dementia or hearing disabilities may lose some of the communication and participation in care‐planning meetings via video conferencing (Graves et al., 2018). Such disabilities are quite common in frail older persons with complex needs for care, which have to be coordinated and planned after hospital discharge.

## 3 METHOD

The study used an inductive qualitative descriptive method (Graneheim & Lundman, 2004). The data collection was conducted through individual interviews with nurses with experience of conducting care‐planning meetings via video conferencing. The ethical considerations advocated by the Helsinki Declaration were taken into account (World Medical Association, 2013).

### 3.1 Participants

A strategic selection of experienced nurses from hospitals, the municipalities and primary care were invited to participate in the interviews. Including nurses from different organizations made it possible to get a variety of responses and study the phenomenon from several perspectives. Eleven nurses (10 female and one male) accepted the invitation to participate. Their professional experience varied between 5 and 23 years, and all participants had experience in care planning via video conferencing. The participants of the study are described in Table 1.

**TABLE 1:** Participants in the study

ID	Role	Professional experience (years)	Gender	Experience in care planning via video conferencing (years)	Organization
1	Nurse	7	Female	5	Primary care
2	Nurse	13	Female	7	Primary care
3	Nurse	10	Female	4	Hospital
4	Nurse	5	Female	2	Hospital
5	Nurse	7	Female	4	Hospital
6	Nurse specialist	19	Female	8	Municipality
7	Nurse specialist	13	Male	2	Municipality
8	Nurse	23	Female	9	Hospital
9	Nurse	23	Female	2	Primary care
10	Nurse specialist	10	Female	2	Primary care
11	Nurse specialist	15	Female	8	Primary care

### 3.2 Collection of data

The data were collected between April 2019 and March 2020. The study thus started before the Covid‐19 pandemic. During the end of the data collection period, the Covid‐19 virus emerged. Therefore, the use of video conferencing increased during this study, in the beginning of the pandemic.

The data collection method was semi‐structured individual interviews with open‐ended questions. An interview guide was used as support so that the same questions were asked at each interview (Kvale, 1996). Follow‐up questions were used to confirm, reflect on and get a deeper understanding of the participants' stories (Polit & Beck, 2016). Each interview lasted between 40 and 90 min; they were conducted at places chosen by the participants, and were recorded and transcribed verbatim.

### 3.3 Data analysis

The interviews were analysed using an inductive method for content analysis, elucidating both manifest and latent content (Graneheim & Lundman, 2004). The inductive analysis involved a back‐and‐forth process between the text and the authors' experiences, and between parts of the text and the whole, which eventually created a new understanding (Elo & Kyngäs, 2008; Hsieh & Shannon, 2005). Manifest content refers to what is directly expressed in the text, and latent content refers to the interpreted meaning of the text. The analysis began with the text being repeatedly read through naïve reading to get a sense of the whole, based on the purpose of the study. Subsequently, the text was divided into meaning units that were condensed and coded. Codes with similar content were grouped into nine sub‐categories that formed three categories, with respect to manifest content. Finally, the overall theme emerged, highlighting the latent content and the underlying meaning of the text (Graneheim et al., 2017; Graneheim & Lundman, 2004). An example of the analysis process is described in Table 2.

**TABLE 2:** Example of the analysis process from meaning unit to category, via condensed meaning unit, code and sub‐category

Meaning unit	Condensed meaning unit	Code	Sub‐category	Category
…I hardly think patients today have any choice because as soon as you do not add the Skype link, some party… will say they are not coming, despite us expressing that this needs to be an in‐person meeting at the hospital	Patients have limited options if the meeting is done in‐person at the hospital or via Skype	The meeting	Adapt to the situation	Person centring

## 4 RESULTS

An overall theme, three categories and nine sub‐categories illuminate how nurses experience person‐centred care planning using video conferencing upon hospital discharge of frail older persons, as presented in Table 3.

**TABLE 3:** Overview of the overall theme, categories and sub‐categories

Latent content	Theme	Person‐centred healthcare planning using video conferencing upon hospital discharge of frail older persons		
Manifest content	Categories	Preparations	Conduct the meeting	Person centring
	Sub‐categories	Different forms of work	Create structure	Listen to the story
		Responsibility to participate	Agree on decisions	Adapt to the situation
		Assess status and efforts	Document jointly	Meet through the screen

### 4.1 Preparations

The informants described the importance of careful preparation of different forms of work in connection with care planning via video, based on a shared responsibility among caregivers to assess the patient's status and the efforts provided to improve the situation.

#### 4.1.1 Different forms of work

The informants pointed out that there was no common conceptual apparatus, which meant that several different terms were used to describe the care‐planning meeting; pre‐meeting, reconciliation meeting, planning meeting, care‐planning meeting and meeting about CIP. The name of the meeting differed depending on whether the patient and relatives were present or not. The informants considered that there was some confusion about the meaning of the various meetings and when during the care process they would take place.

Book, what we call, a reconciliation meeting, we don't call it care‐planning meeting because it should be kept short… when the patient has returned home, a CIP at home should be booked… But a lot is done at this reconciliation meeting with the hospital via Skype [video conferencing] and then we don't need to have a CIP meeting (9).

#### 4.1.2 Responsibility to participate

The informants pointed out that participation increased when care planning was carried out via video conferencing. The informants mentioned their own responsibilities in bringing about effective meetings, but emphasized the responsibilities of the other care providers more. When, for example, the need for home care was identified, primary care would become responsible to make this contact. The informants pointed out the ongoing discussions between care providers about the need for home care, as this need governs whether a nurse, physiotherapist or occupational therapist should participate from the municipality or primary care. It emerged that responsibility meant participating in the care‐planning meeting, so when the patient had home care, primary care did not have to participate.

If you miss essential professionals who need to be there, then the meeting is half done, then you may still have to do it again, but primary care is not included if the patient is enrolled in home health care (8).

#### 4.1.3 Assess status and efforts

The informants described the importance of being able to follow the status of the patient during the hospital stay in order to correctly assess and plan the interventions. It was also considered important that information about what efforts had been provided to the patient before the hospitalization was documented in the common IT system. Another important aspect in assessing status and efforts was that participating parties knew what their own and other organizations could offer to the patient and relatives in terms of care. One of the most difficult parts of assessing status and efforts, as highlighted by the informants, was assessing whether and when a CIP would be implemented. When it was decided that a CIP would be carried out at home, the informants considered that it could be difficult to have time to do it, if the patient risked being readmitted to hospital soon.

A little difficult to say what is a CIP and what is care planning… usually they end up back in the hospital before you have time to do any CIP again so… it is back‐and‐forth (11).

### 4.2 Conduct the meeting

The informants emphasized the importance of creating a structure, agreeing on decisions and documenting, to effectively conduct video‐conference care‐planning meetings.

#### 4.2.1 Create structure

The informants pointed out the importance of having a chairman for the meeting, who allows everyone to speak. The chairman was also expected to be the rapporteur. They indicated that there was uncertainty and lack of knowledge about the meaning of this role. They believed that it was important to have knowledge about their own technical equipment, and use a meeting template and checklists to create a good structure for the meeting. Care‐planning meetings via video were considered to be shorter and more structured than physical meetings. This was also an important reason as to why the patients were able to participate in the meetings.

The video meetings become more focused and a little shorter… there are many who can't manage to attend [a meeting] for an hour (5).

#### 4.2.2 Agree on decisions

The informants emphasized the importance of agreeing on decisions about which efforts would be relevant to apply after discharge. The informants pointed out the ongoing discussion among care providers about the need for rehab and home care for patients. The informants also highlighted other problems in connection with discharge, such as disagreement with the doctors' decisions, whether the patient was really ready to be discharged or when the discharge was to be planned.

How can you think the patient is ready for discharge … can't participate in the care planning even once because they are so bad… but you can't keep on arguing with a patient with home care and rehab (11).

#### 4.2.3 Document jointly

The informants expressed disappointment that the patients' documentations were not regularly updated by all care providers. Lack of time and knowledge about what to document and where, were considered to be the reasons. To remedy the problem, they urged each other to document relevant information in the common IT system. It emerged that those who did not attend a meeting could communicate their assessments and efforts in the IT system either before or after the meeting.

You have to be in the system all the time as well… so it takes quite a lot of time (3).

### 4.3 Person centring

The informants said that care planning via video presupposes that care‐providing parties listen to the person and adapt the situation to meet in a person‐centred manner through a monitor.

#### 4.3.1 Listen to the story

Listening to the patient's story is one of the important, basic principles of person‐centred care, the informants said. It was important to be able to balance between allowing the patient to speak about his/her life and deciding when the story became too long. During the meeting, it was important to respectfully direct the conversation towards the planning, and at the same time maintain the interaction with the patient.

When you can, direct the conversation to this planning that has to be done, but the interaction with the patient is the most important (5).

#### 4.3.2 Adapt to the situation

The informants pointed out that video conferencing as a meeting format has become a first choice over physical meetings in care planning, without giving the patient much choice. They considered that since the care‐planning meeting put the patient and relatives in a vulnerable situation, it was important to provide information, show the patient respect and make the patient truly involved in the decision‐making. They said that when there was an established relationship with the patient, it was easier to adapt the situation and make the patient more involved in the video conference. It was also easier to know how the patient worked and what she or he wanted help with. The informants described that the patients would be so happy to recognize someone on the other side of the screen that it seemed as if they forgot that they were not sitting in the same room.

It is a vulnerable position as a patient to sit there… oh, then they appreciate that they recognize me… hey, [xxx] now I recognize you (10).

#### 4.3.3 Meet through the screen

According to the informants, it was more difficult to meet the patient as a person via the screen, and they experienced a form of distance when the proximity was lost in the video meeting. Some aspects were absent, such as eye contact, head movements, gaze, handshake and how the person's physical appearance. Vision, hearing and cognitive impairments made the video meeting more difficult, but the informants believed that the same difficulties could also exist in physical meetings. They considered that the screen did not directly affect the outcome of the meeting.

Don't feel that what is lost compared to a physical meeting have any effect on the result (1).

## 5 DISCUSSION

Healthcare planning via video conferencing is experienced differently by nurses from different healthcare organizations. Healthcare planning via video conferencing is also different from in‐person meetings, from a person‐centred care perspective. We have identified nurses' experiences of healthcare transition from hospital to the person's home, coordination and collaboration among healthcare professionals, as well as a person‐centred work practice in healthcare planning.

The study shows that video conferencing as a meeting format has become a first choice over physical meetings when planning care for frail older persons who need coordinated care interventions at home after discharge from hospital, even before the Covid‐19 pandemic. When a frail older person leaves the hospital, it is important to assess care needs, and plan and coordinate care interventions together with care providers, the older person and relatives. Well‐functioning information transfer in an online journal is a necessary tool based on current legislation. But for this to work, coordination of care providers' professional resources and interprofessional collaboration is required (Larsson et al., 2019; Pullon et al., 2016).

The results show that multidisciplinary video conferencing is increasingly conducted without the patient and relatives, despite the fact that care and nursing needs are discussed on the basis of the patient's current health status. These video meetings aim to reach a consensus among care‐providing parties about which care measures and interventions may be relevant after discharge. The development thus indicates that fewer meetings are held together with patients and relatives during the hospital stay. The idea is to transfer information through a common specific online patient record that is available to the care providers. The question is how care‐providing parties can ensure a person‐centred approach after discharge from the hospital. This is where nurses have a unique coordinating function. Ekman et al. (2011) state that person‐centring means that the patient is treated as an equal party and the partnership is compared to a team consisting of healthcare staff, patient and relatives. The person's influence and participation in decisions about care interventions constitute cornerstones in person‐centred care, which are difficult to maintain during discharge if the patient is not present. As video conferencing makes meetings more challenging, due to difficulties in meeting through the screen, the person centredness could be impaired.

This study points to several aspects of complex conditions in care transitions using video conferencing. The results show that there is frustration among nurses that frail older persons often are too ill to be sent home. When care planning is carried out with staff who lack knowledge about how the person really feels, patient safety is at risk. This risk is even larger when using video conferencing. Research shows that caregiving parties believe that they provide information relevant to the context, while the other parties claim the opposite. This shows that there is a lack of trust between the parties, which is needed in order to achieve effective collaboration. The use of video conferencing further contributes to decreasing the trust. Larsson et al. (2019) explain that the reason for this is that there is an organizational hubris among care‐providing parties, which means that everyone feels better than the others in terms of collaboration for the patient group. In the video‐conferencing era of care‐planning meetings, this calls for another approach to delivering person‐centred care, to compensate for the shortcomings that can emerge due to technical limitations. The results of this study show that the written documentation provided more suggestions for interventions than the description of the person's current status. Jobe (2020) found in his study that there is confusion about what and how to document in the different parts of the care plan. The information is of low quality and information is also often missing. In addition to the documentation being incomplete, the information transfer does not work either, which makes it difficult to have effective care‐planning meetings via video and ensure good care at home. In addition, care staff risk being perceived as unprofessional by patients and relatives when they lack knowledge about the patient's health condition and needs at the care‐planning meeting, according to Jobe (2020).

The results show that there is a lack of requirements from the management regarding the nurses' competence and education. Nurses currently do not need any form of formal education at the advanced level to work with care planning via video conferencing. Knowledge of assessment and collaboration to achieve coordinated care was also requested. Research shows that nursing staff trained at the advanced level excel in comparison with undergraduates, thanks to their deeper knowledge about independently assessing and planning care measures to avoid “unnecessary” hospitalizations (Glassman, 2016; Jobe, 2020).

In this study, it emerged that when care planning is carried out via video conferencing, a barrier is created that requires the nurses to strengthen the older person by creating an interaction that is maintained throughout the meeting (Graves et al., 2018). It also emerged that the nurses sometimes lack ability to listen to the older persons' stories, when they want to talk about something other than what the professionals want. Person centring is a strategy that promotes the experience of interaction in care settings, according to McCormack and McCance (2006). The fact that older persons and relatives know the care staff from before is described in the results as a strength when the care planning is carried out via video conferencing. The recognition factor stimulates stories about care occasions when care‐providing parties already have information about the person. The recognition factor stimulates the patient's stories about previous care occasions, which helps care‐providing parties to get a rich picture of the person's situation.

The study also shows that there are no established routines for how older persons should manage their care plans themselves, where joint decisions and care interventions are documented, after the video conference. At present, older persons and relatives have to manage this themselves. However, it could be a security, at least for the relatives, to receive written information, if the older person does not have the strength to read her−/himself. The informants expressed fear that there would be too much written information for the older person to manage in connection with discharge. This should be set against the joint responsibility to document in the care plan, which constitutes a cornerstone of person‐centred care according to Ekman et al. (2011).

Hedqvist and Svensson (2019) point out that lack of information about the structure and content of the video conference makes it difficult for older persons and relatives to prepare for the meeting. They experience insecurity and feel neglected in a situation unknown to them. Our study also shows that older persons have limited opportunities to really influence care design: the scope for action is too small and they experience that they are not given any choices during the video conference itself. Facchinetti et al. (2021) believe that this is because the care staff need to inform, involve and prepare the older persons for the discharge, so that they can handle their condition at home without feeling abandoned. Sinclair et al. (2020) found that functioning coordinated planning of care interventions before discharge has become more important in connection with the outbreak of the Covid‐19 pandemic in 2020. The pandemic has thus accelerated the development of the digitalization of collaborative working methods such as coordinated planning of care interventions via video conferencing. Silsand et al. (2021) show that the use of video conferencing offers opportunities to use healthcare professionals' time more efficiently, reduces travel times and improves the exchange of information across healthcare providers' professional boundaries.

## 6 CONCLUSIONS

In summary, this study concludes that it is complex to create a sustainable way of working for nurses with regard to planning and coordinating care interventions. It is difficult to maintain person‐centred care unless the older person or relatives are involved in the care and in the care planning throughout the entire process. Although a cornerstone of person‐centred care is meeting face‐to‐face, video conferencing can be seen as a complement in care for coordination and follow‐up of care and treatment. More research is needed to study different methods using digital tools for improved coordination of care for frail older persons with complex care needs.

## ETHICAL STATEMENT

The study has been approved by the Swedish Ethical Review Authority [Approval number: 932‐18].

## Data Availability

Data available on request due to privacy/ethical restrictions.
